# The long-term fate of permafrost peatlands under rapid climate warming

**DOI:** 10.1038/srep17951

**Published:** 2015-12-09

**Authors:** Graeme T. Swindles, Paul J. Morris, Donal Mullan, Elizabeth J. Watson, T. Edward Turner, Thomas P. Roland, Matthew J. Amesbury, Ulla Kokfelt, Kristian Schoning, Steve Pratte, Angela Gallego-Sala, Dan J. Charman, Nicole Sanderson, Michelle Garneau, Jonathan L. Carrivick, Clare Woulds, Joseph Holden, Lauren Parry, Jennifer M. Galloway

**Affiliations:** 1 water@leeds, School of Geography, University of Leeds, LS2 9JT, United Kingdom; 2 School of Geography, Archaeology and Palaeoecology, Queen’s University Belfast, BT7 1NN, United Kingdom; 3 Geography, College of Life and Environmental Sciences, University of Exeter, EX4 4RJ, United Kingdom; 4 Department of Geosciences and Natural Resource Management, Center for Permafrost (CENPERM), University of Copenhagen, DK-1350, Denmark; 5 Geological Survey of Sweden, Uppsala, Sweden; 6 Département de Géographie and GEOTOP, Université du Québec à Montréal, Montréal, Québec, Canada; 7 School of Interdisciplinary Studies, Dumfries Campus, University of Glasgow, Rutherford/McCowan Building, Crichton, Dumfries, DG1 4ZL, United Kingdom; 8 Natural Resources Canada/Ressources naturelles Canada, Geological Survey of Canada/Commission géologique du Canada, Calgary, Alberta, T2L 2A7, Canada

## Abstract

Permafrost peatlands contain globally important amounts of soil organic carbon, owing to cold conditions which suppress anaerobic decomposition. However, climate warming and permafrost thaw threaten the stability of this carbon store. The ultimate fate of permafrost peatlands and their carbon stores is unclear because of complex feedbacks between peat accumulation, hydrology and vegetation. Field monitoring campaigns only span the last few decades and therefore provide an incomplete picture of permafrost peatland response to recent rapid warming. Here we use a high-resolution palaeoecological approach to understand the longer-term response of peatlands in contrasting states of permafrost degradation to recent rapid warming. At all sites we identify a drying trend until the late-twentieth century; however, two sites subsequently experienced a rapid shift to wetter conditions as permafrost thawed in response to climatic warming, culminating in collapse of the peat domes. Commonalities between study sites lead us to propose a five-phase model for permafrost peatland response to climatic warming. This model suggests a shared ecohydrological trajectory towards a common end point: inundated Arctic fen. Although carbon accumulation is rapid in such sites, saturated soil conditions are likely to cause elevated methane emissions that have implications for climate-feedback mechanisms.

Twenty-first century climatic warming is projected to be greatest in high-latitude areas of the Northern Hemisphere. IPCC AR5 climate models project that global mean surface temperatures are likely to increase by 0.3 °C to 4.8 °C by the end of the 21^st^ century relative to 1986-2005, with a very high confidence that the Arctic region will warm more rapidly. Projected temperature increases over the Arctic land region have central estimates of 1.9 °C (RCP2.6), 3.9 °C (RCP4.5), 4.5 °C (RCP6.0) and 7.5 °C (RCP8.5)^1^. The implications for ecosystem structure and carbon budgets at high latitudes are likely to be of global importance through biosphere-climate feedbacks that have the potential to either accelerate or dampen the global warming effect^2^. Zones of permafrost have retreated rapidly poleward in recent decades, evidenced by the widespread development of degradation features such as thaw lakes^3^, increased active layer thickness^4^ and in some locations the complete disappearance of permafrost^5^,^6^.

Given their relatively small global areal extent, permafrost peatlands are disproportionately important to the future of global-scale ecosystem-climate feedbacks. Organic-rich permafrost peat stores approximately 277 Pg of carbon (C)^7^, equivalent to 14% of the global soil C store^8^. Until recently this huge soil C store has been rendered effectively inert, protected from decomposition by lethargic microbial activity in frozen soil conditions. The prospect of widespread permafrost thaw leaves this C store vulnerable to rapid decomposition, with a huge reciprocal global warming potential from increased fluxes of greenhouse carbon gases (GHGs) – chiefly CH_4_ from waterlogged soil conditions – to the atmosphere^9^. However, this global warming effect may be partially compensated or even outweighed entirely by increased CO_2_ sequestration through newly-invigorated ecosystem productivity and peat accumulation^10^.

Contemporary GHG flux rates from degrading permafrost peatlands, and their relationships to highly localised water-table and temperature measurements, have only been intensively monitored since the 1990s^6^. A dearth of palaeoecological studies into the response of permafrost peatlands to climatic change during the instrumental period (i.e., the last 100–150 years) leaves the future of degrading permafrost peatlands, and their likely feedbacks to the global climate system, highly unclear.

The Abisko region of northern Sweden (Supplementary material 1) is an area characterised by currently degrading permafrost peat^11^,^12^. Abisko has experienced rapid warming during the twentieth century^13^; mean annual air temperature exceeded the 0 °C threshold around AD 2000 leaving the region beyond the climatic envelope that can sustain permafrost. Climate model projections suggest continued marked temperature increases in the near future (Supplementary material 2). Active-layer deepening and increase in surface wetness through thawing of permafrost are both coincident with the sharp temperature rise in the last ~30 years (Fig. 1).

Distinct forms of degraded permafrost peatlands can be identified in Abisko, despite similar climatic conditions across the region. These include partially collapsed palsas and peat plateaux, thermokarst lakes, and Arctic fens and bogs that no longer contain permafrost (Supplementary material 1). However, it is unclear whether these distinct forms represent divergent trajectories for degrading permafrost peatlands, or stages along a pathway towards a common end-point. The answer to this question has important implications for the future of permafrost peatlands and their global-scale ecosystem-climate feedbacks. Earlier research on permafrost peatlands suggested cyclical models of palsa development under steady climates^14^. Such an explanation for the distinct permafrost forms at our study area seems unlikely to hold given that the entire region has now surpassed the 0 °C threshold and continues to warm, making refreezing and development of new palsas all but impossible. We reconstruct the recent ecohydrological and carbon dynamics of currently degrading Abisko peatlands to assess the likely future trajectories of Northern Hemisphere permafrost peat in response to future warming in the arctic and subarctic.

We analysed peat cores from i) a desiccating permafrost bog; ii) an area of peatland that has recently collapsed due to permafrost degradation; and iii) an Arctic fen, currently devoid of permafrost (Supplementary material 1, 3-7). All three of our study sites have become drier over the last century (Figs 2 and 3). However, two sites (the collapsed peatland and Arctic fen) show a subsequent abrupt shift to wetter conditions. In the Arctic fen this wet shift tracks the temperature increase of the latter twentieth century (Fig. 1), whereas the collapsed peatland is influenced by water-table fluctuations in the surrounding fen. The desiccating bog exhibits a strong drying trend and has not undergone any rapid shift to wetter conditions.

Although the number of observations is limited, correlation analysis (Supplementary material 8) illustrates that in the case of the permafrost-free Arctic fen there are significant negative correlations between temperature data for several months throughout the year and reconstructed water-table depth. This indicates the site has become wetter due to thawing permafrost elsewhere in the catchment. The water-table depth reconstruction from the collapsed peatland is largely uncorrelated with instrumental temperature variables providing further evidence that the site has now passed a threshold beyond which its hydrology is controlled by autogenic mechanisms rather than climate. The desiccating bog is strongly linked to climate, where water-table depth exhibits positive correlations with temperature for several months of the year. This site has become drier due to temperature-driven increases in evapotranspiration.

Despite some similarities in hydrology, the three sites exhibit contrasting carbon accumulation (CA) regimes. CA rates are typically much higher in the upper peat profile in most peatland systems because full decomposition has yet to take place; however, the substantial differences in CA regime between the three sites here indicate a change in CA dynamics through time. In the desiccating bog CA has remained extremely low due to large decomposition losses^cf.^^15^. The collapsed peatland has a very high CA, seemingly prompted by early twentieth century warming; since the collapse, CA rates have become mostly disconnected from climate and exhibit variable temporal behaviour which we interpret as allogenic (climate) and autogenic (internal feedbacks) controls competing for dominance. In the Arctic fen CA has increased sharply in recent years, likely due to increased productivity from higher temperatures^16^ and reduced decomposition in anoxic, saturated peat.

We propose five distinct phases along a trajectory of degradation for permafrost peatlands (Fig. 4). We contend that genuinely pristine permafrost peatlands (Phase 1) are no longer present in our study region because mean annual temperature has been above 0 °C for more than a decade. The second stage (Phase 2; desiccating) is characterised by drying of surficial peat due to higher temperatures, leading to greater evapotranspirative losses, desiccation of the peat surface, slow lowering of the water table and high levels of decomposition. The system is driven by allogenic climatic forcing in Phase 2. Phase 3 represents a threshold of rapid change: continued drying leads to peat shrinkage and the peat surface begins to crack (very commonly observed in the field – Fig. 4, Phase 3 photo), increasing thermal connectivity between the atmosphere and what remains of the permafrost. The result is a collapsed peatland (Phase 4) due to runaway degradation of permafrost, causing rapid collapse of the peatland and saturation with thaw water. In the final stage (Phase 5; Arctic fen) the peatland is devoid of permafrost; it is now influenced by surface and groundwater flow into the system from adjacent areas and local hydrochemistry^17^. This final stage has the potential for large carbon sequestration through newly invigorated productivity and rapid peat accumulation (Fig. 3f); however, elevated methane fluxes also seem likely owing to saturated soils^9^,^18^,^19^.

Although autogenic mechanisms have dominated ecosystem dynamics during certain periods of permafrost degradation, persistent warming has eventually forced inevitable collapse, followed by re-invigorated productivity and peat accumulation. This is in contrast to commonly held concerns about catastrophic loss of the peatland C stock under future climate change^20^. The temporal limit of ongoing monitoring campaigns provides only a partial record of the response of permafrost peatlands to recent warming. Palaeoecological studies such as ours and investigations of longer-term changes during the Holocene provide important baseline information over longer timescales that allows a fuller understanding of the fate of degrading permafrost peatlands.

## Methods

**Figure 2:**
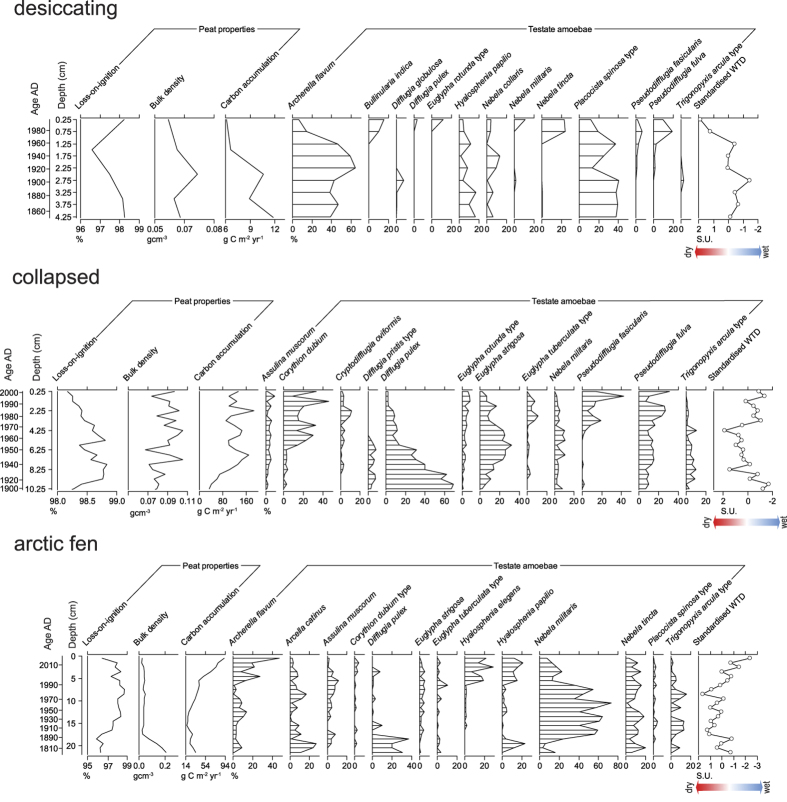
Peat property and testate amoeba data from the three study sites. Chronological determinations are from the age models are shown (see Supplementary material 7). Standardised water-table reconstructions are illustrated (see Supplementary material 5).

We identified three different peatlands in the Abisko region in different states of permafrost decay, despite being subject to the same climate: 1) desiccating bog albeit with largely intact permafrost; 2) recently thawed and partially collapsed area of peatland surrounded by fen; and 3) Arctic fen with no current permafrost and abundant thaw pools (Supplementary material 3 and 4). We collected peat cores from the Arctic fen and desiccating bog using a Russian corer^21^. Refer to^12^ for information on sampling of the collapsed peatland. In the laboratory we carried out bulk density and loss-on-ignition analyses following standard methods^22^. Carbon accumulation was calculated following^23^. We analysed testate amoebae in each core following^24^ (Fig. 2), and the transfer function of^17^ was used for water-table depth reconstruction. Water-table depth data were standardised following^25^. The chronology of each core was based on ^210^Pb, AMS radiocarbon, spheroidal carbonaceous particles and tephrochronology (Supplementary material 6) and age-depth models were constructed using linear interpolation between dates (Supplementary material 7). We compiled available data on active layer thickness and instrumental climate data to compare with the peat-based data. For more detailed information on methods refer to Supplementary material 5.

## Additional Information

**How to cite this article**: Swindles, G. T. *et al.* The long-term fate of permafrost peatlands under rapid climate warming. *Sci. Rep.* **5**, 17951; doi: 10.1038/srep17951 (2015).

## Supplementary Material

Supplementary Information

## Figures and Tables

**Figure 1:**
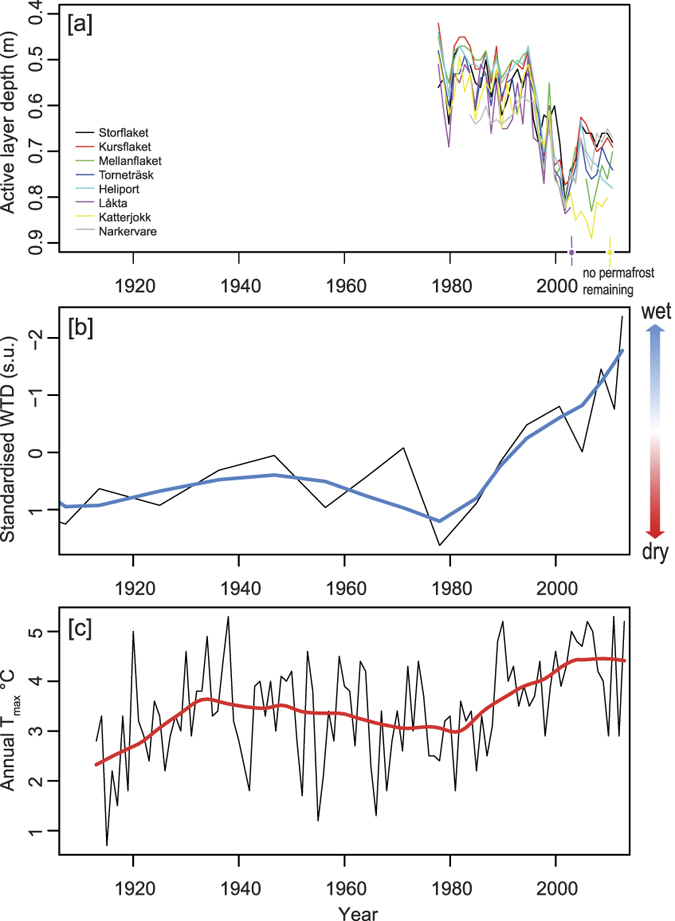
Recent changes in the Abisko region (**a**) deepening of the active layer since the 1980s (data from^4^,^26^). (**b**) Rapid shift to wetter conditions in an Arctic fen starting at ~1980 (this study, reconstructed using testate amoebae – see Fig. 2), blue line shows a locally-weighted scatterplot smoothing function; s.u. = standardised water table units^25^. (**c**) Annual maximum temperature from Abisko showing two distinct periods of warming in the twentieth century (see Supplementary material 1 and 2); the red line shows a locally-weighted scatterplot smoothing function.

**Figure 3:**
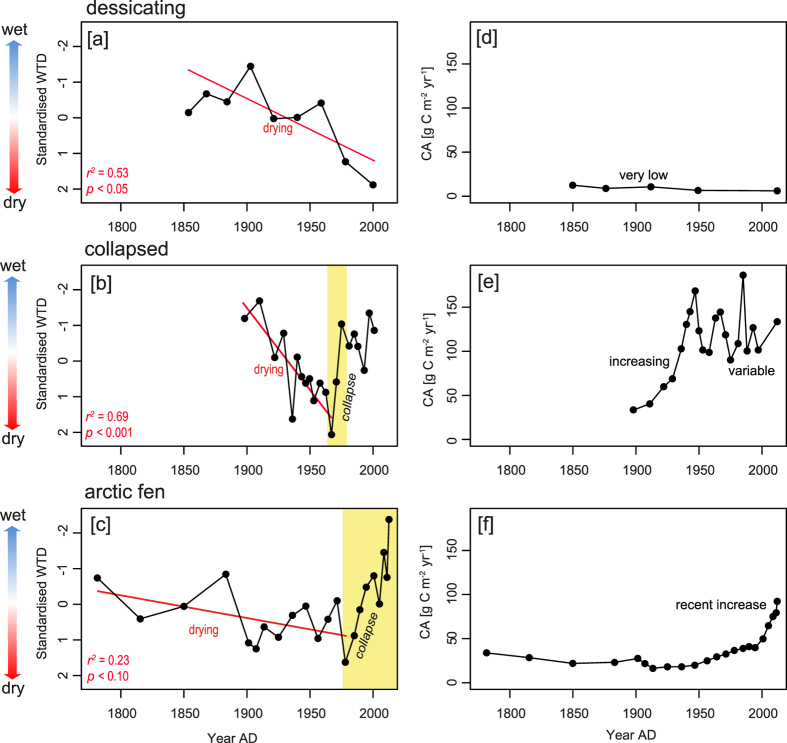
(**a**–**c**) Standardised water-table reconstructions based on testate amoeba analysis from the three study sites. All sites show a marked drying trend until the latter twentieth century; however, the collapsed peatland and Arctic fen show a subsequent rapid shift to wetness. Linear regression statistics for the drying trends in each site are shown. (**d**–**f**) Annotated carbon accumulation data from the three sites.

**Figure 4:**
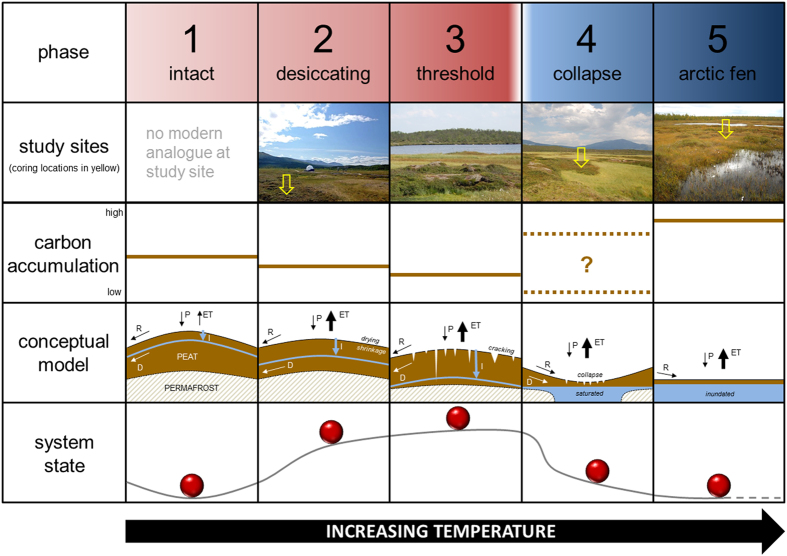
Five phase model for degrading permafrost peatlands in response to increasing temperature. Each column corresponds to one of five distinct phases, identified in the top row. Symbols in the conceptual model represent the following hydrological fluxes: R = runoff; D = shallow drainage; P = precipitation; ET = evapotranspiration; I = infiltration. The bottom row illustrates ecohydrological stability of the system using a ball-and-cup analogy^27^. The first phase (‘intact’) represents the only stable basin of attraction before climate-driven change alters the system state.

## References

[b1] Christensen J. H. *et al.* Climate Phenomena and their Relevance for Future Regional Climate Change in Climate Change 2013: The Physical Science Basis. Contribution of Working Group I to the Fifth Assessment Report of the Intergovernmental Panel on Climate Change 1–6, pp. 1217–1308 (2013). 10.1017/CBO9781107415324

[b2] Hartmann D. L., Tank a. M. G. K. & Rusticucci M. IPCC Fifth Assessment Report, Climate Change 2013: The Physical Science Basis. IPCC **AR5**, 31–39 (2013).

[b3] Jorgenson M. T. & Osterkamp T. E. Response of boreal ecosystems to varying modes of permafrost degradation. Canadian Journal of Forest Research 35, 2100–2111 (2005).

[b4] Åkerman H. J. & Johansson M. Thawing permafrost and thicker active layers in sub-arctic Sweden. Permafr. Periglac. Process. 19, 279–292 (2008).

[b5] Sollid J. L. & Sørbel L. Palsa bogs as a climate indicator: examples from Dovrefjell, southern Norway. Ambio 27, 287–291 (1998).

[b6] Johansson M., Christensen T. R., Akerman H. J. & Callaghan T. V. What determines the current presence or absence of permafrost in the Torneträsk region, a sub-arctic landscape in northern Sweden? Ambio 35, 190–197 (2006). 16944644 10.1579/0044-7447(2006)35[190:wdtcpo]2.0.co;2

[b7] Tarnocai C. *et al.* Soil organic carbon pools in the northern circumpolar permafrost region. Global Biogeochem. Cycles 23, GB2023 (2009).

[b8] IPCC. IPCC Special Report: Land use, land-use change, and forestry Summary for Policymakers. 1–9 (2000). 10.2277/0521800838

[b9] Moore T. R. & Roulet N. T. Methane flux: Water table relations in northern wetlands. Geophys. Res. Lett. 20, 587 (1993).

[b10] Klein E. S., Yu Z. & Booth R. K. Recent increase in peatland carbon accumulation in a thermokarst lake basin in Southwestern Alaska. Palaeogeogr. Palaeoclimatol. Palaeoecol. 392, 186–195 (2013).

[b11] Malmer N. & Wallén B. Peat Formation and Mass Balance in Subarctic Ombrotrophic Peatland around Abisko, Northern Scandinavia. Ecol. Bull. 45, 79–92 (1996).

[b12] Kokfelt U. *et al.* Ecosystem responses to increased precipitation and permafrost decay in subarctic Sweden inferred from peat and lake sediments. Glob. Chang. Biol. 15, 1652–1663 (2009).

[b13] Callaghan T. V. *et al.* A new climate era in the sub-Arctic: Accelerating climate changes and multiple impacts. Geophys. Res. Lett. 37 (2010).

[b14] Zuidhoff F. S. & Kolstrup E. Palsa Development and Associated Vegetation in Northern Sweden. Arctic, Antarctic, and Alpine Research 37, 49–60 (2005).

[b15] Fenner N. & Freeman C. Drought-induced carbon loss in peatlands. Nat. Geosci. 4, 895–900 (2011).

[b16] Charman D. J. *et al.* Climate-related changes in peatland carbon accumulation during the last millennium. Biogeosciences 10, 929–944 (2013).

[b17] Swindles G. T. *et al.* Evaluating the use of testate amoebae for palaeohydrological reconstruction in permafrost peatlands. Palaeogeogr. Palaeoclimatol. Palaeoecol. 424, 111–122 (2015a).

[b18] McCalley C. K. *et al.* Methane dynamics regulated by microbial community response to permafrost thaw. Nature 514, 478–481 (2014). 25341787 10.1038/nature13798

[b19] Treat C. C. *et al.* Temperature and peat type control CO_2_ and CH_4_ production in Alaskan permafrost peats. Glob. Chang. Biol. 20, 2674–2686 (2014). 24616169 10.1111/gcb.12572

[b20] Ise T., Dunn A. L., Wofsy S. C. & Moorcroft P. R. High sensitivity of peat decomposition to climate change through water-table feedback. Nature Geoscience 1, 763–766 (2008).

[b21] De Vleeschouwer F., Chambers F. M. & Swindles G. T. Coring and sub-sampling of peatlands for palaeoenvironmental research. Mires Peat 7, 1–10 (2010).

[b22] Chambers F. M., Beilman D. W. & Yu Z. Methods for determining peat humification and for quantifying peat bulk density, organic matter and carbon content for palaeostudies of climate and peatland carbon dynamics. Mires Peat 7, 1–10 (2011).

[b23] Tolonen K. & Turunen J. Accumulation rates of carbon in mires in Finland and implications for climate change. The Holocene 6, 171–178 (1996).

[b24] Booth R. K., Lamentowicz M. & Charman D. J. Preparation and analysis of testate amoebae in peatland palaeoenvrionmental studies. Mires Peat 7, 1–7 (2010).

[b25] Swindles G. T. *et al.* Testing peatland water-table depth transfer functions using high-resolution hydrological monitoring data. Quat. Sci. Rev. 120, 107–117 (2015b).

[b26] Akerman H. J. Active layer monitoring, Abisko area, Sweden. Active layer monitoring, Abisko area, Sweden. In: International Permafrost Association, Data and Information Working Group, comp. Circumpolar Active-Layer Permafrost System (CAPS), version 1.0. CD-ROM available from National Snow and Ice Data Center, NSIDC (1998). At http://nsidc.org/data/docs/fgdc/ggd207_activlayer_sweden/. Accessed 20^th^ June 2015.

[b27] Scheffer M. *et al.* Catastrophic shifts in ecosystems. Nature 413, 591–596 (2001). 11595939 10.1038/35098000

