# Supplementary material for: The long-term fate of permafrost peatlands under rapid climate warming
Source: Sci Rep. 2015 Dec 9;5:17951. doi: 10.1038/srep17951 (PMC4673699; doi:10.1038/srep17951)
Supplement: Supplementary Information [file srep17951-s1.pdf]

The long-term fate of permafrost peatlands under rapid climate warming

Supplementary material

Graeme T. Swindles[1], Paul J. Morris[1], Donal Mullan[2], Elizabeth Watson[1], T. Edward Turner[1], Tom Roland[3], Matthew J. Amesbury[3], Ulla Kokfelt[4], Kristian Schoning[5], Steve Pratte[6], Angela Gallego-Sala[3], Dan J. Charman[3], Nicole Sanderson[3], Michelle Garneau[6], Jonathan L. Carrivick[1], Clare Woulds[1], Joseph Holden[1], Lauren Parry[7] and Jennifer M. Galloway[8]

[1]School of Geography, University of Leeds, LS2 9JT, United Kingdom, g.t.swindles@leeds.ac.uk [Corresponding author]

[2]School of Geography, Archaeology and Palaeoecology, Queen's University Belfast, BT7 1NN, United Kingdom

[3]Geography, College of Life and Environmental Sciences, University of Exeter, EX4 4RJ, United Kingdom

[4]Department of Geosciences and Natural Resource Management, Center for Permafrost (CENPERM), University of Copenhagen, DK-1350, Denmark

[5]Geological Survey of Sweden, Uppsala, Sweden

[6]Département de Géographie and GEOTOP, Université du Québec à Montréal, Montréal, Québec, Canada

[7]School of Interdisciplinary Studies, Dumfries Campus, University of Glasgow, Rutherford/McCowan Building, Crichton, Dumfries, DG1 4ZL, United Kingdom

[8]Natural Resources Canada/Ressources naturelles Canada, Geological Survey of Canada/Commission géologique du Canada, Calgary, Alberta, T2L 2A7, Canada

Supplementary material 1 - Study area

The climate of Abisko, Northern Sweden is considerably drier than other locations at similar latitudes (Tveito et al., 2000; Yang et al., 2012). Lying in the rain shadow of the Norwegian mountains, mean annual precipitation is just 332 mm (1981-2010), which is relatively low compared with neighbouring locations such as Narvik, Norway (< 100 km away) where annual precipitation reaches around 800mm (Callaghan et al., 2010). The seasonal precipitation variability is high, with lowest mean totals of 46 mm during the spring months and highest mean totals of 136 mm during summer (1981-2010). Temperatures are relatively mild for a location inside the Arctic Circle, with a long-term mean annual temperature of -0.6°C for 1913-2006 (Akerman and Johansson, 2008), and a recent thirty year average of 0.1°C from 1981-2010. For this time period, mean winter and summer temperatures have been -9.3°C and 10.1°C respectively. These relatively mild temperatures are due to the relatively close proximity to the Atlantic Ocean (<100 km away). Long-term climate records for Abisko Scientific Research Station from 1913 reveal an abrupt warming phases from the late 1930s to the early 1940s, and a more recent shift from the mid-1970s to present (Callaghan et al., 2010; Yang et al., 2012). Mean annual temperatures have increased by 2.5°C since 1913, with an accelerated increase of ~1.5°C since 1974 (Callaghan et al., 2010). Based on the most recent available thirty-year averaging period (1984-2013), the mean annual temperature now frequently exceeds 0°C - an important threshold temperature above which permafrost is particularly vulnerable (Smith and Riseborough, 1983).

Lowland areas of the Abisko region are characterised by extensive peatlands. These include ombrotrophic bogs, peat plateaux, arctic fens and palsa mires (Figure 2). Palsa mires are particularly common features of the Abisko region. Palsa mires are frost-heaved peat mounds with a permanently frozen core and are characteristic of the circumpolar discontinuous permafrost zone (Lundqvist, 1969; Seppälä, 1972; Nelson et al., 1992; Seppälä, 1997; Gurney, 2001). There is much concern over the future of palsa mires in a warmer world as their frozen core needs to survive the heat of the summer season (Seppälä, 2006). As these peatlands are very sensitive to climate change there has been much discussion regarding the decreasing areal extent and degradation of palsas due to climate warming, and the effect this will have on C dynamics (Matthews et al., 1997; Zuidhoff and Kolstrup, 2000; Luoto and Seppala, 2002, 2003; Luoto et al., 2004a; Luoto et al., 2004b; Payette et al., 2004). There has been a lot of peatland research in the Abisko region (e.g. Malmer and Wallén, 1996; Dorrepaal et al., 2009; Kokfelt et al., 2009; Swindles et al., 2015a). The Stordalen mire complex is probably one of the most researched and instrumented peatlands in the world. Recent studies have shown there to be deepening of the active layer and accelerated decay of permafrost at Stordalen due to increased temperature and snow depth, which has caused enlargement of minerotrophic areas and decline of relatively dry ombrotrophic peatland (Christensen et al., 2004; Malmer et al.,

2005; Johansson et al., 2006). Two of our cores came from the Stordalen mire complex – one from a desiccating bog, albeit with largely intact permafrost; and one from an area of collapsed peatland (0.4 km apart). Our third core came from a different peat complex, an Arctic fen, 59 km to the southeast. This site has no permafrost currently – although there are peatlands with permafrost < 1km away. The site is marked as a palsa in the Quaternary Geology Map of Sweden (Geological Survey of Sweden) based on field mapping that was carried out in 1970. We therefore contend that the site lost its permafrost sometime after 1970.

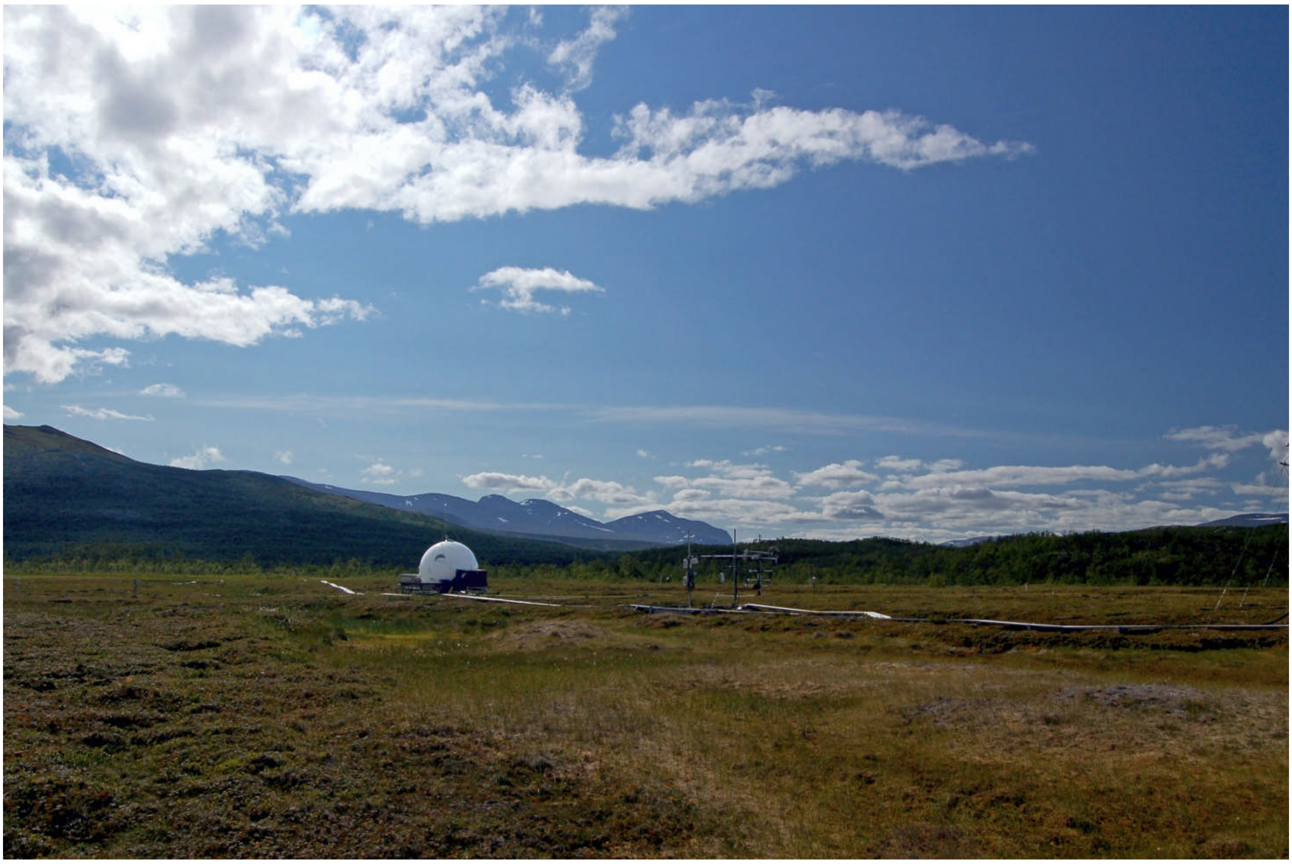

🡅 Desiccating bog (Phase 2)

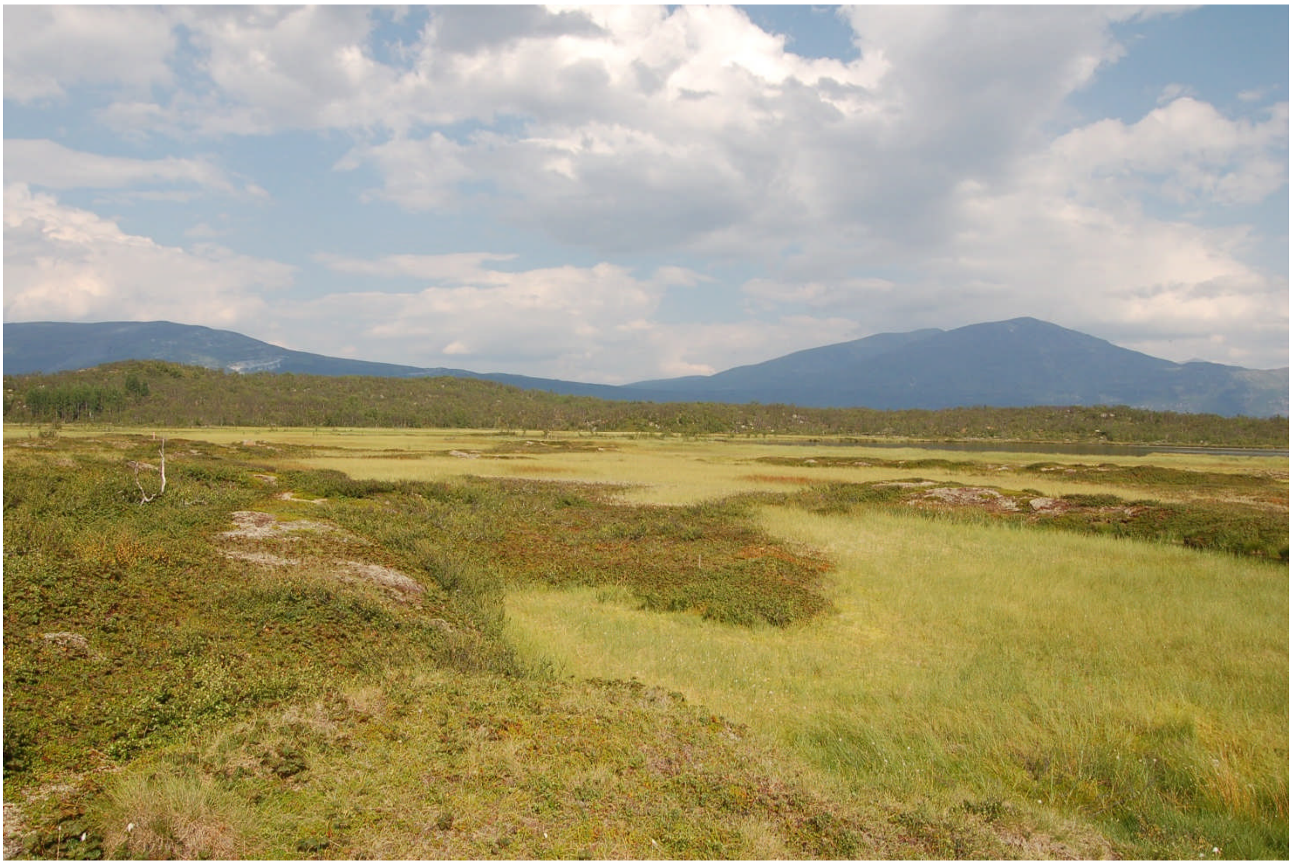
🡹 Areas of collapsed peatland (Phase 4)

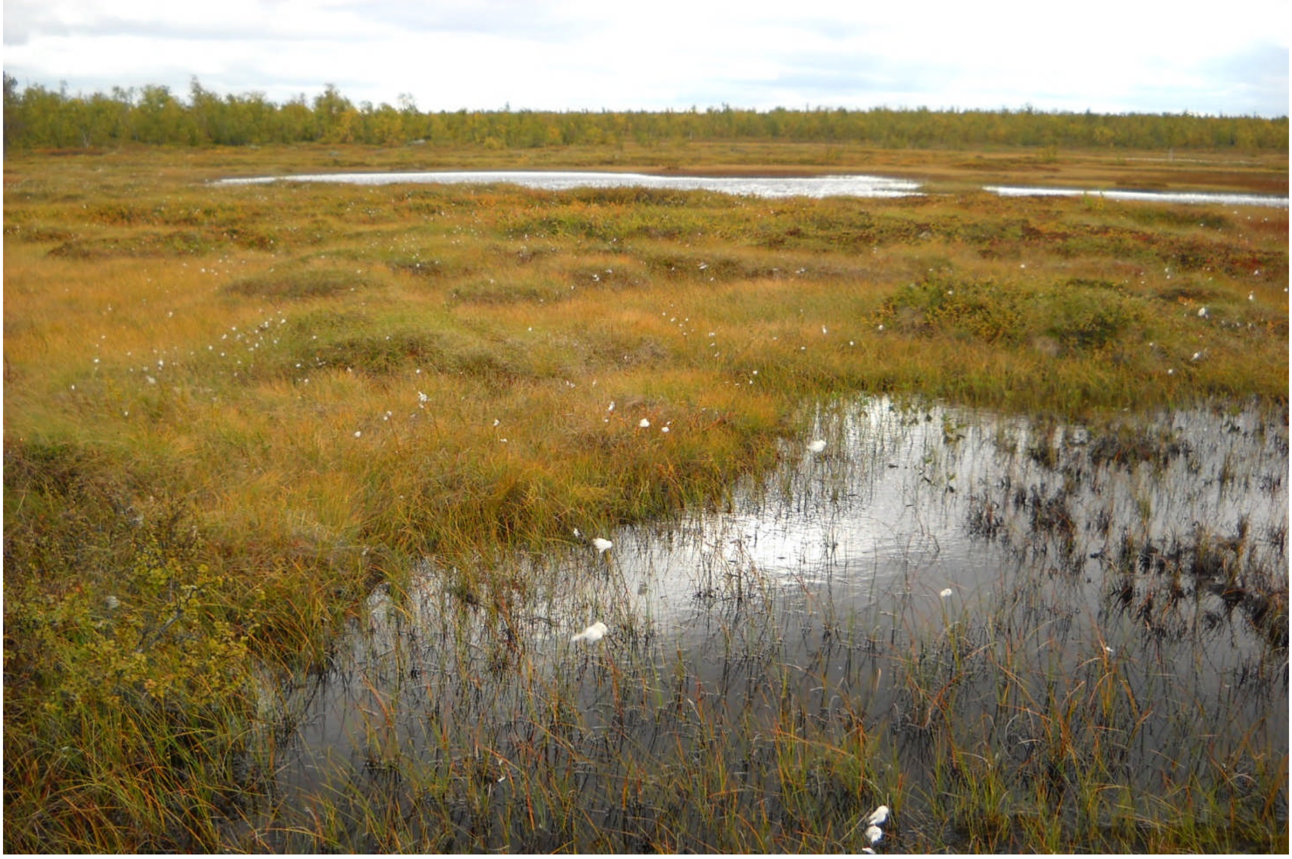
🡹 Arctic fen (Phase 5)

Supplementary material 2 – Climate change in Abisko

Observed mean annual temperatures illustrating the large change in the 1980s. The red line is a locally weighted scatterplot smoothing function. A continuous wavelet analysis is also shown illustrating the breakdown of significant cyclical components starting in the 1970s. The black line highlights periodicities significant at the 95% level.

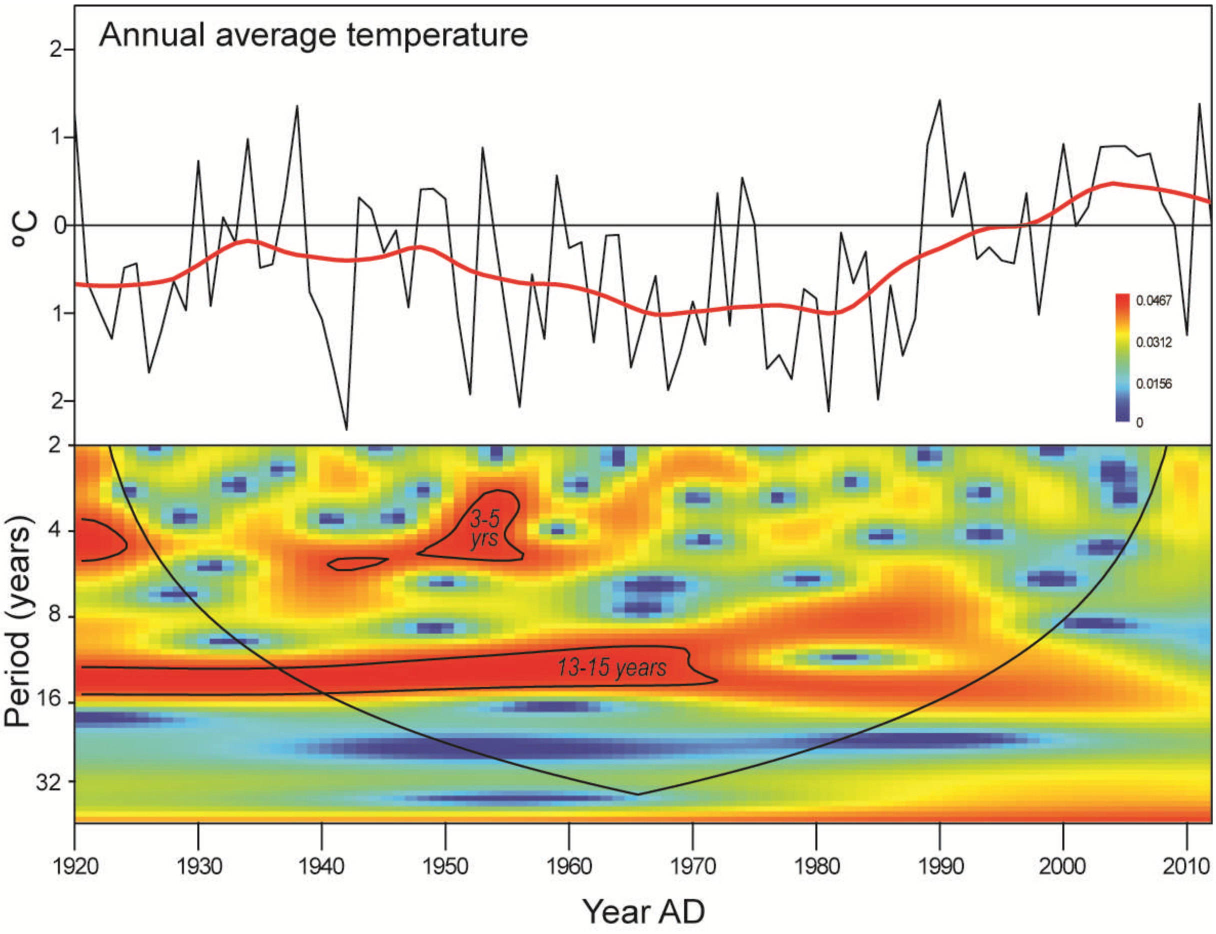

Observed mean annual temperatures (1913-2012) in black; and modelled mean annual temperatures, including future projections (2001-2100) in red at Abisko Scientific Research Station. The modelled temperatures are statistically downscaled from the Canadian Centre for Climate Modelling and Analysis Coupled Global Climate Model 3 (CGCM3), driven by the A1B and A2 emissions scenarios. The red line represents the mean of 20 ensembles (grey lines).

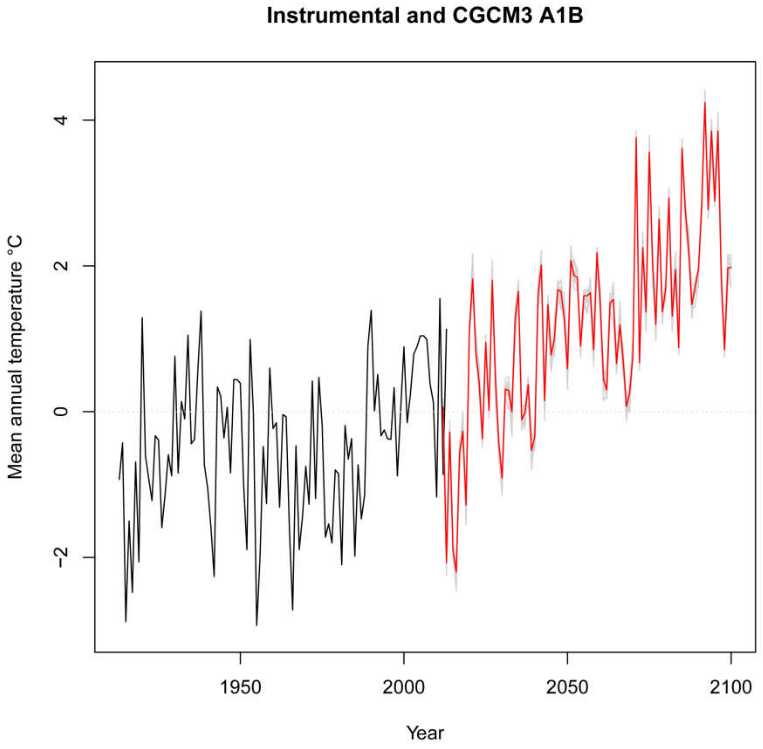

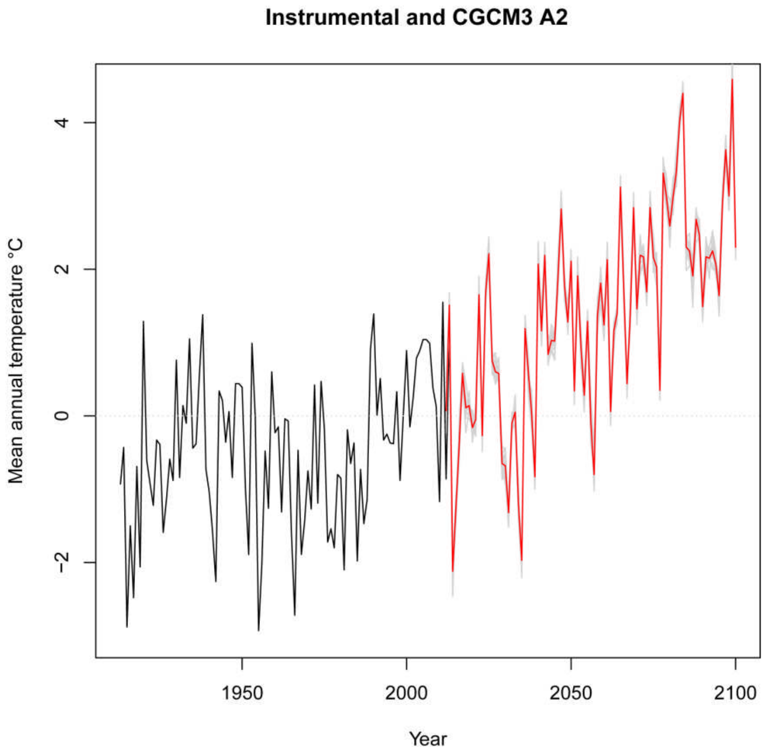

Supplementary material 3 - Regional map with site locations

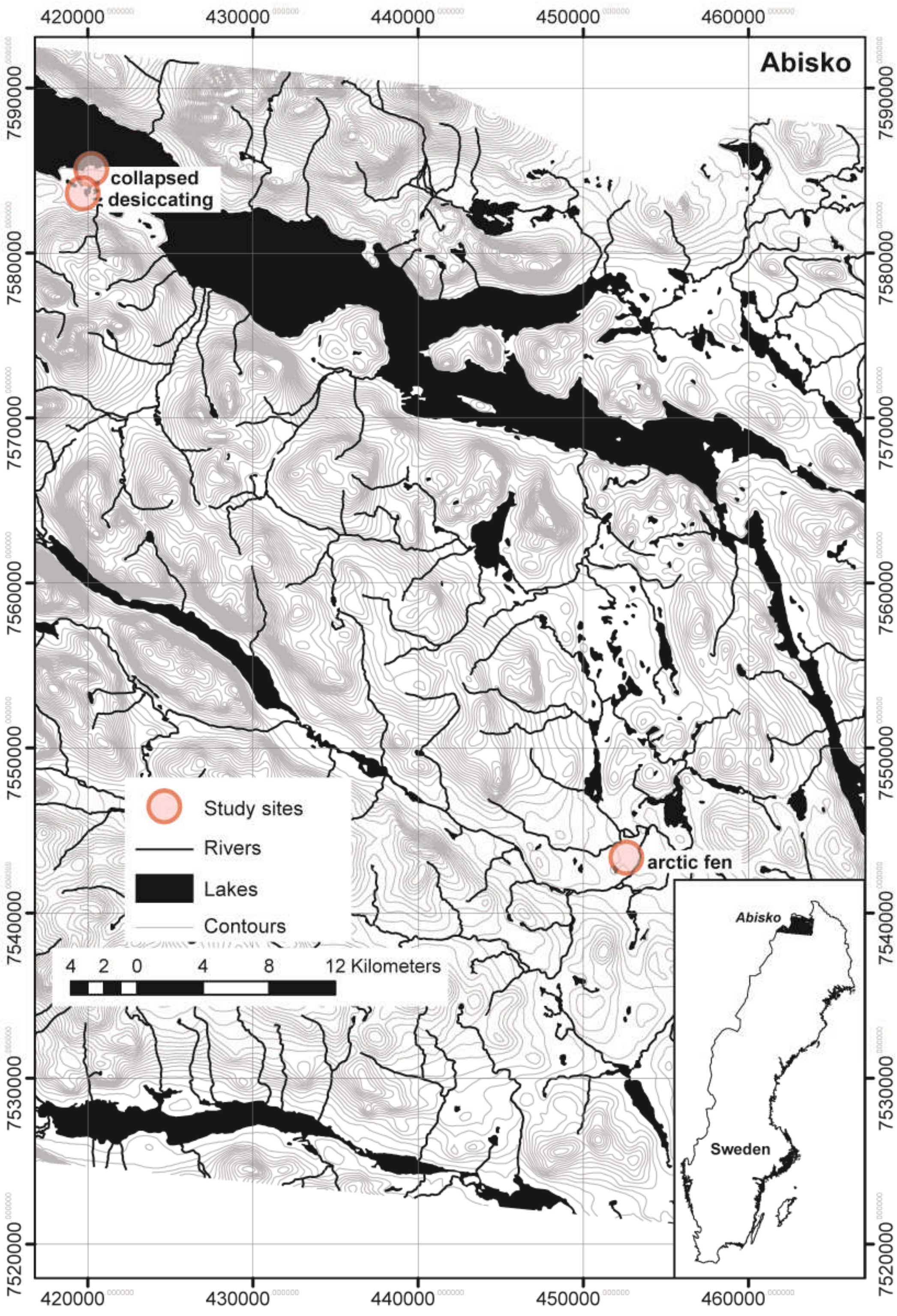

The map was created by the authors in ArcGIS 10.2, using contours and landcover polygons at 1:50,000 scale by Landmateriert (Swedish Mapping authority, http://kso.lantmateriet.se/). Our site locations were positioned in the field with a handheld Garmin etrex Summit global positioning system (GPS).

Supplementary material 4 - Study sites

We identified three different peatlands in the Abisko region each at different phases of permafrost decay:

1) Desiccating bog (western peatland at Stordalen);

N 68°21'23.0"

E 19°02'39.0"

DD 68.356361, 19.044111

2) Area of collapsed peatland surrounded by fen (north-eastern peatland at Stordalen);

N 68˚21'25.4"

E 19˚03'14.0"

DD 68.357055, 19.053888

3) Arctic fen with no current permafrost and abundant thaw pools (Arctic fen – 3.5 km NW of Krovik).

N 67°57'24.0"

E 19°59'11.4"

DD 67.956666, 19.9865

Site characteristics (water table measured in auger holes; negative values indicate standing water)

**Site name**	**Peatland type**	**Water-table depth range (cm) (on day of sampling)**	**pH range**
3.5 km NW of Krovik	Poor arctic fen	-1 to 29 (*n* = 7)	3.24 - 4.21
Stordalen I and II	Palsa-peat plateaux complex and fen	-7 to 50 (*n* = 40)	2.99 - 3.80

Supplementary material 5 - Methods

0.5m-long peat cores were extracted from each site. We selected the coring location following site walkovers to ensure that it was representative of the site as a whole in terms of physical features, hydrology and vegetation composition. The representatively of the stratigraphy was also checked through in-the-field analysis of a number of trial cores across the site. The cores were wrapped in plastic wrap and aluminium foil, and stored at 4˚C prior to analysis. The cores were extracted using a Russian corer following De Vleeschouwer et al. (2010) and wrapped in plastic wrap and aluminium foil and shipped to the University of Leeds. At the University of Leeds the cores were stored in refrigeration at 4°C prior to analysis. The samples were subsequently ignited in a muffle furnace at 450°C for 8 hours to determine loss-on-ignition values (Schulte and Hopkins, 1996). In the laboratory we carried out bulk density and loss-on-ignition analyses following standard methods (Chambers et al., 2010) and carbon (C) accumulation rates were calculated from the age-depth model peat accumulation rates, bulk density and C content following Tolonen and Turunen (1996). Calculated C accumulation rates are 'apparent' rates of carbon accumulation (due to potential further decay), thus they represent the balance between production and decay (Clymo et al 1998). Productivity is particularly important in determining actual rates of carbon accumulation in Arctic peatlands (Gao and Couwenberg, 2015).

Testate amoebae were extracted using a modified version of Booth et al. (2010). Peat samples were placed in boiling water for 15 minutes and shaken. Extracts were passed through a 300 μm sieve, back-sieved at 15 μm and allowed to settle before sub-samples were used to make slides for microscopy. 100 to 200 amoebae were counted and identified to species level or 'type' in each sample using high-power transmitted light microscopy at 200 to 400x magnification. Identification was aided with reference to several sources (Leidy, 1879; Penard, 1902; Cash and Hopkinson, 1905; Cash and Hopkinson, 1909; Cash et al., 1915; Deflandre, 1936; Grospietsch, 1958; Corbet, 1973; Ogden and Hedley, 1980; Charman et al., 2000; Meisterfeld, 2001a; Meisterfeld, 2001b). The taxonomy used was a modified version of Charman et al. (2000), where some 'type' groupings are split to the species level (e.g. *Phryganella acropodia* and *Difflugia globulosa* were split out of *Cyclopyxis arcelloides* type when possible). This is an established, pragmatic taxonomic approach for analysis of Holocene peat samples (Charman et al., 2000). The testate amoebae in the collapsed area of peatland have been published previously (Kokfelt et al., 2009); however, the temperature European transfer function used in this paper has been superseded by a more appropriate model for permafrost peatlands (Swindles et al., 2015a). The transfer function of Swindles et al. (2015a) was applied to each core for water-table reconstruction from the subfossil data. The transfer function model used is based on WA.tol with inverse deshrinking. Water-table depth reconstructions were standardised following Swindles et al. (2015b). All statistical analyses were carried out in R version 3.0.2 (R-Core-Team, 2014) using the packages vegan (Oksanen, 2012), rioja (Juggins, 2012).

Peat samples were extracted and submitted to the following laboratories for ^{210}Pb analysis: Geotop-UQAM Radiochronology laboratory, Montreal, Canada (desiccating bog – alpha spectrometry); Gamma Dating Center, Copenhagen, Denmark (collapsed area of peatland – gamma spectrometry); and University of Exeter, United Kingdom (Arctic fen – alpha spectrometry). Constant rate of supply (CRS) models were used to assign ages to the ^{210}Pb data (Appleby, 2001) and ^{137}Cs was used to verify the age model in the case of the collapsed peatland. In the desiccating bog only the first two centimetres yielded ages that fit within ^{210}Pb limits; therefore, ^{14}C, spheroidal carbonaceous particles (SCPs) and tephra was used to help date this sequence. Above-ground remains of plant macrofossils were extracted from the profile and submitted to the DirectAMS laboratory (Bothell, Washingston) for AMS radiocarbon dating. The dates were calibrated using IntCal13 (Reimer et al., 2013).

The core from the desiccating bog was analysed for tephra using the quick burn technique (Hall and Pilcher, 2002; Swindles et al., 2010). After burning, the tephra was sieved at 15 µm in an ultrasonic bath for 20 minutes to remove fine siliceous material, rinsed with deionised water, and the coarse fraction mounted onto slides. Tephra shard counts were conducted at 200 × magnification on a standard Leica binocular microscope. Following detection of the peak tephra shard concentration, tephra was extracted for geochemical analysis following the density separation method of (Blockley et al., 2005). The peat sample was sieved between 80 and 10 µm sieve sizes. Further extraction was conducted using various densities of Fast Float heavy liquid. A cleaning float of 2.0 g cm^{-3} was used to remove organic material a further float at of 2.2 g cm^{-3} was also required to remove fine siliceous material. Finally tephra was floated off at 2.5 g cm^{-3} and rinsed thoroughly with deionised water. Samples were mounted onto glass slides using EpoThin resin, ground to expose the shards (cf. Dugmore et al., 1992) and polished to a 0.25 µm finish. Analysis was conducted by EPMA at the University of Edinburgh. Analysis setup followed the method of Hayward (2012), all analyses were conducted with a beam diameter of 5 µm, 15 kV and beam currents of 2 nÅ (Na, Mg, Al, Si, K, Ca, Fe) or 80 nÅ (P, Ti, Mn). Secondary glass standards, rhyolite (Lipari) and basalt (BCR-2G) were analysed before and after runs of unknown glass standard analyses. Analysis of SCPs was carried out following Swindles (2010). We assigned an age of AD 1875 to the first appearance of SCPs in the record based on the closest precisely-dated lake record (Sannajärvi), which is to the north of our field sites on the Finland-Swedish border (69°05′N, 20°52′E) (Ruppel et al., 2013). Age-depth models were produced using linear interpolation between dates (using maximum probability in the case of the ^{14}C dates).

Data on active layer thickness and instrumental climate data were compiled from online sources:

http://polar.se/en/abisko-naturvetenskapliga-station/vaderdata/

http://www.gwu.edu/~calm/data/webforms/s2_f.html#_DATA

Supplementary material 6 – Geochemical identification of tephra layer in the desiccating bog (Stordalen 22-24 cm) to Hekla 1158

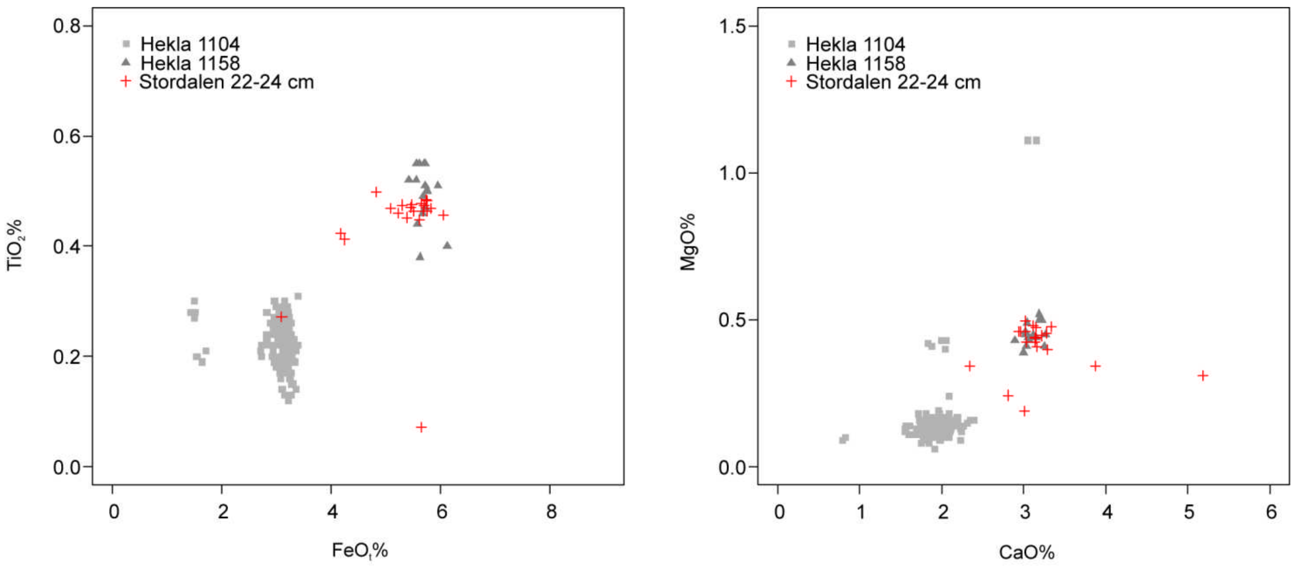

Supplementary material 7 – Age-depth models (all dates are ^{210}Pb unless otherwise stated).

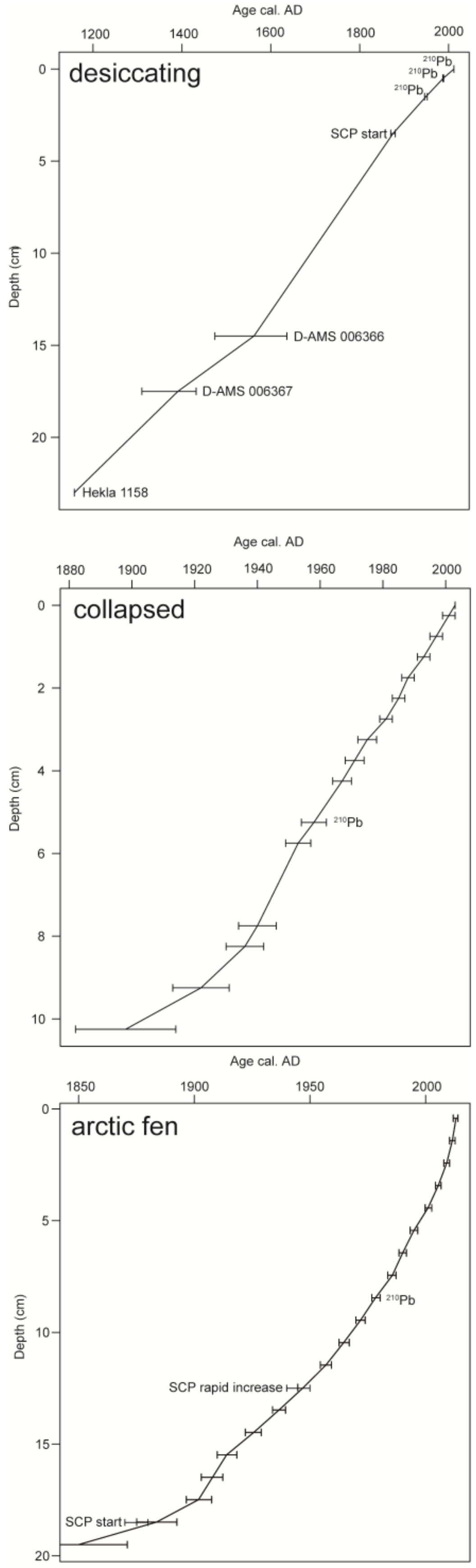

Supplementary material 8 – Correlation matrix of water-table reconstructions against instrumental climate data. TN = Mean annual temperature; TX = Maximum annual temperature; PP = Total annual precipitation; NAO = North Atlantic Oscillation index. The analysis was carried out for 1, 3 and 5 year windows of the instrumental climate data.

desiccating (phase 2)

	Jan				Feb				Mar				Apr			
	TN	TX	PP	NAO	TN	TX	PP	NAO	TN	TX	PP	NAO	TN	TX	PP	NAO
Exact Yr	-0.25	-0.40	-0.14	0.03	-0.64	-0.67	-0.70	-0.67	0.01	0.05	0.61	-0.15	-0.25	-0.33	-0.80	-0.55
5 Yr	-0.06	-0.08	0.18	-0.52	-0.95	-0.97	0.09	-0.17	0.17	0.21	0.70	-0.11	0.65	0.56	0.05	-0.42
					*	**										
3 Yr	0.17	0.11	0.08	-0.36	-0.89	-0.92	0.15	-0.31	0.10	0.18	0.94	-0.03	0.21	0.13	-0.27	-0.42
					*	*					*					

	May				Jun				Jul				Aug			
	TN	TX	PP	NAO	TN	TX	PP	NAO	TN	TX	PP	NAO	TN	TX	PP	NAO
Exact Yr	-0.40	-0.34	-0.11	-0.16	0.20	-0.04	-0.02	-0.21	0.48	0.36	0.89	-0.48	0.18	-0.07	-0.01	-0.71
											*					
5 Yr	0.71	0.82	0.23	0.27	0.92	0.92	0.13	0.17	0.06	-0.29	0.62	-0.14	0.43	0.42	-0.93	-0.62
					*	*									*	
3 Yr	0.52	0.51	0.26	0.12	0.81	0.81	0.15	0.19	0.48	-0.38	0.89	-0.07	0.22	0.04	-0.49	-0.50
											*					

	Sep				Oct				Nov				Dec				Ann			
	TN	TX	PP	NAO	TN	TX	PP	NAO	TN	TX	PP	NAO	TN	TX	PP	NAO	TN	TX	PP	NAO
Exact Yr	-0.73	-0.19	0.55	0.05	-0.15	-0.33	0.41	-0.57	0.69	0.60	0.40	0.18	-0.87	-0.97	-0.16	-0.25	-0.48	-0.64	0.31	-0.70
														**						
5 Yr	0.45	0.34	-0.04	0.28	0.17	0.08	0.76	-0.30	0.51	0.47	0.62	0.30	0.58	0.64	0.31	0.48	0.39	0.41	0.47	-0.19
3 Yr	0.39	0.26	-0.22	0.35	0.44	0.20	0.68	-0.32	0.94	0.78	0.44	0.28	0.58	0.60	0.21	0.45	0.34	0.28	0.61	-0.23
									*											

collapsed (phase 4)

	Jan				Feb				Mar				Apr			
	TN	TX	PP	NAO	TN	TX	PP	NAO	TN	TX	PP	NAO	TN	TX	PP	NAO
Exact Yr	-0.11	-0.18	-0.34	-0.09	-0.13	-0.14	-0.04	-0.14	-0.07	-0.05	-0.06	-0.04	0.02	-0.01	-0.01	0.00
5 Yr	-0.09	-0.11	0.03	-0.17	-0.30	-0.39	-0.24	-0.52	-0.40	-0.43	0.43	-0.04	-0.15	0.02	0.20	0.16
								*								
3 Yr	-0.15	-0.23	-0.07	0.03	-0.36	-0.45	-0.38	-0.43	-0.39	-0.38	0.24	0.06	-0.08	0.06	-0.05	0.24

	May				Jun				Jul				Aug			
	TN	TX	PP	NAO	TN	TX	PP	NAO	TN	TX	PP	NAO	TN	TX	PP	NAO
Exact Yr	-0.09	-0.06	-0.33	-0.01	-0.04	-0.02	0.07	0.03	-0.10	-0.09	-0.08	-0.10	-0.05	-0.04	-0.06	-0.12
5 Yr	-0.44	-0.55	-0.55	0.03	-0.17	-0.09	-0.06	0.55	-0.11	0.04	-0.26	0.14	0.19	0.34	-0.25	-0.20
		*	*					**								
3 Yr	-0.34	-0.35	-0.43	-0.01	-0.02	-0.01	0.01	0.49	-0.13	-0.04	-0.20	-0.04	-0.18	-0.08	-0.10	-0.46
								*								

	Sep				Oct				Nov				Dec				Ann			
	TN	TX	PP	NAO	TN	TX	PP	NAO	TN	TX	PP	NAO	TN	TX	PP	NAO	TN	TX	PP	NAO
Exact Yr	-0.05	-0.05	-0.13	-0.06	-0.10	-0.07	-0.29	0.06	0.03	0.03	0.08	-0.04	0.00	-0.06	-0.25	-0.11	-0.07	-0.06	-0.21	-0.05
5 Yr	0.07	-0.04	-0.04	0.31	-0.09	-0.02	-0.31	0.27	0.16	0.12	-0.37	-0.32	-0.04	-0.14	-0.07	-0.29	-0.26	-0.25	-0.38	-0.09
3 Yr	-0.13	-0.25	-0.09	0.27	-0.18	-0.10	-0.25	0.19	0.41	0.35	0.04	-0.25	0.29	0.19	0.09	-0.41	-0.24	-0.29	-0.23	-0.08

arctic fen (phase 5)

	Jan				Feb				Mar				Apr			
	TN	TX	PP	NAO	TN	TX	PP	NAO	TN	TX	PP	NAO	TN	TX	PP	NAO
Exact Yr	-0.29	-0.33	0.44	0.00	-0.20	-0.10	-0.28	-0.14	0.14	0.21	0.13	0.53	-0.22	-0.35	-0.22	-0.05
												*				
5 Yr	-0.46	-0.45	0.27	0.06	0.17	0.21	-0.35	-0.22	-0.53	-0.49	-0.33	0.44	-0.45	-0.43	-0.14	0.18
									*							
3 Yr	-0.47	-0.45	0.45	-0.12	-0.22	-0.10	-0.16	-0.21	-0.19	-0.16	0.04	0.38	-0.26	-0.24	0.21	0.39

	May				Jun				Jul				Aug			
	TN	TX	PP	NAO	TN	TX	PP	NAO	TN	TX	PP	NAO	TN	TX	PP	NAO
Exact Yr	-0.52	-0.61	-0.34	0.04	-0.45	-0.41	-0.17	0.18	0.26	0.45	-0.17	0.15	-0.59	-0.58	-0.03	-0.19
	*	*											*	*		
5 Yr	-0.39	-0.51	-0.22	-0.15	-0.58	-0.58	-0.53	0.37	-0.22	-0.04	-0.34	0.36	-0.54	-0.36	-0.08	0.09
		*			*	*	*						*			
3 Yr	-0.42	-0.60	-0.16	0.08	-0.45	-0.43	-0.45	0.20	0.12	0.18	-0.37	0.24	-0.38	-0.30	-0.11	-0.03
		*														

	Sep				Oct				Nov				Dec				Ann			
	TN	TX	PP	NAO	TN	TX	PP	NAO	TN	TX	PP	NAO	TN	TX	PP	NAO	TN	TX	PP	NAO
Exact Yr	-0.66	-0.69	-0.03	0.27	-0.21	-0.16	-0.03	-0.03	-0.15	-0.27	-0.34	0.07	-0.48	-0.44	-0.44	-0.09	-0.49	-0.54	-0.33	0.15
	***	***																*		
5 Yr	-0.67	-0.64	-0.27	0.31	-0.58	-0.54	-0.47	0.23	-0.45	-0.50	-0.33	0.25	-0.44	-0.44	-0.02	-0.15	-0.61	-0.62	-0.57	0.34
	***	**			*	*				*							*	*	*	
3 Yr	-0.52	-0.58	-0.18	0.53	-0.40	-0.40	-0.27	0.33	0.10	-0.06	-0.27	0.42	-0.25	-0.26	0.02	0.20	-0.46	-0.51	-0.43	0.45
	*	*		*														*		

* Significant at 0.05 ** Significant at 0.01 *** Significant at 0.005

Exact Yr = correlations established between WTD and meteorological data from corresponding year established by age-depth model
3 Yr = correlations established between WTD and meteorological data from 3 year average around the corresponding year established by age-depth model
5 Yr = correlations established between WTD and meteorological data from 5 year average around the corresponding year established by age-depth model

Light blue = r values of -0.4 to -0.59
Dark blue = r values of -0.6 to -1
Light red = r values of 0.4 to 0.59
Dark red = r values of 0.6 to 1

Supplementary material 9 – References

Akerman, H.J., Johansson, M., 2008. Thawing permafrost and thicker active layers in sub-arctic Sweden. Permafrost Periglac 19, 279-292.

Blockley, S., Pyne-O'Donnell, S.D.F., Lowe, J., Matthews, I., Stone, A., Pollard, A.M., Turney, C.S.M., Molyneux, E.G.A., 2005. A new and less destructive laboratory procedure for the physical separation of distal glass tephra shards from sediments. Quat Sci Rev 24, 1952-1960.

Callaghan, T.V., Bergholm, F., Christensen, T.R., Jonasson, C., Kokfelt, U., Johansson, M., 2010. A new climate era in the sub-Arctic: Accelerating climate changes and multiple impacts. Geophys Res Lett 37, 14705.

Christensen, T.R., 2014. Climate science: Understand Arctic methane variability. Nature 509, 279–281.

Clymo, R.S., Turunen, J., Tolonen, K., 1998. Carbon accumulation in peatland. Oikos 81, 368–88.

Dorrepaal, E., Toet, S., van Logtestijn, R.S.P., Swart, E., van de Weg, M.J., Callaghan, T.V., Aerts, R., 2009. Carbon respiration from subsurface peat accelerated by climate warming in the subarctic. Nature 460, 616-619.

Dugmore, A.J., Newton, A.J., Sugden, D.E., Larsen, G., 1992. Geochemical stability of fine-grained silicic Holocene tephra in Iceland and Scotland. J Quaternary Sci 7, 173–183.

Gao, Y., Couwenberg, J., 2015. Carbon accumulation in a permafrost polygon peatland: steady long-term rates in spite of shifts between dry and wet conditions. Glob Change Biol 21, 803–815.

Gurney, S.D., 2001. Aspects of the genesis, geomorphology and terminology of palsas: perennial cryogenic mounds. Prog Phys Geog 25, 249-260.

Hall, V.A., Pilcher, J.R., 2002. Late-Quaternary Icelandic tephras in Ireland and Great Britain: detection, characterization and usefulness. Holocene 12, 223-230.

Hayward, C., 2012. High spatial resolution electron probe microanalysis of tephras and melt inclusions without beam-induced chemical modification. Holocene 22, 119-125.

Johansson, T., Malmer, N., Crill, P.M., Friborg, T., Akerman, J.H., Mastepanov, M., Christensen, T.R., 2006. Decadal vegetation changes in a northern peatland, greenhouse gas fluxes and net radiative forcing. Global Change Biol 12, 2352-2369.

Kokfelt, U., Rosen, P., Schoning, K., Christensen, T.R., Forster, J., Karlsson, J., Reuss, N., Rundgren, M., Callaghan, T.V., Jonasson, C., Hammarlund, D., 2009. Ecosystem responses to increased precipitation and permafrost decay in subarctic Sweden inferred from peat and lake sediments. Global Change Biol 15, 1652-1663.

Lundqvist, J., 1969. Earth and ice mounds: a terminological discussion. in: Péwé, T.L. (Ed.), The Periglacial Environment. Past and Present: McGill-Queen's University Press, Montreal, pp. 203-215.

Luoto, M., Fronzek, S., Zuidhoff, F.S., 2004a. Spatial modelling of palsa mires in relation to climate in northern Europe. Earth Surf Proc Land 29, 1373-1387.

Luoto, M., Heikkinen, R.K., Carter, T.R., 2004b. Loss of palsa mires in Europe and biological consequences. Environ Conserv 31, 30-37.

Luoto, M., Seppala, M., 2002. Modelling the distribution of palsas in Finnish lapland with logistic regression and GIS. Permafrost Periglac 13, 17-28.

Luoto, M., Seppala, M., 2003. Thermokarst ponds as indicators of the former distribution of palsas in Finnish lapland. Permafrost Periglac 14, 19-27.

Malmer, N., Johansson, T., Olsrud, M., Christensen, T.R., 2005. Vegetation, climatic changes and net carbon sequestration in a North-Scandinavian subarctic mire over 30 years. Global Change Biol 11, 1895-1909.

Malmer, N., Wallén, B., 1996 Peat formation and mass balance in subarctic ombrotrophic peatlands around Abisko, northern Scandinavia. Ecological Bulletins 45, 79–92.

Matthews, J.A., Dahl, S.O., Berrisford, M.S., Nesje, A., 1997. Cyclic development and thermokarstic degradation of palsas in the mid-Alpine zone at Leirpullan, Dovrefjell, southern Norway. Permafrost Periglac 8, 107-122.

Nelson, F.E., Hinkel, K.M., Outcalt, S.I., 1992. Palsa-scale frost mounds in: Dixon, J.C., Abrahams, A.D. (Eds.), Periglacial geomorphology. John Wiley & Sons, Chichester, pp. 305-325.

Payette, S., Delwaide, A., Caccianiga, M., Beauchemin, M., 2004. Accelerated thawing of subarctic peatland permafrost over the last 50 years. Geophys Res Lett 31, L18208.

Ruppel, M., Lund, M.T., Grythe, H., Rose, N.L., Weckström, J., Korhola, A., 2013. Comparison of spheroidal carbonaceous particle data with modelled atmospheric black carbon concentration and deposition and air mass sources in Northern Europe, 1850–2010. Advances in Meteorology, vol. 2013, Article ID 393926, 15 pages.

Seppälä, M.T., 1972. The term “palsa”. Zeitschrift für Geomorphologie 16, 463.

Seppälä, M., 1997. Introduction to the periglacial environment in Finland. Bulletin of the Geological Society of Finland 69, 73-86.

Seppälä, M., 2006. Palsa mires in Finland, in: Lindholm, T., Heikkilä, R. (Eds.), The Finnish Environment 23/2006, pp. 155–162.

Smith, M.W., Riseborough, D.W., 1983. Permafrost sensitivity to climate change, 4th Int. Permafrost Conference. Nat. Academy Press, Washington, DC, Fairbanks, AK, pp. 1178-1183.

Swindles, G.T., 2010. Dating recent peat profiles using spheroidal carbonaceous particles (SCPs). Mires and Peat 7, 1-10.

Swindles, G.T., De Vleeschouwer, F., Plunkett, G., 2010. Dating peat profiles using tephra: stratigraphy, geochemistry and chronology. Mires and Peat 7, 1-9.

Swindles, G.T., Amesbury, M., Turner, T.E., Carrivick, J.L., Woulds, C., Raby, C., Mullan, D., Roland, T.P., Galloway, J., Parry, L., Kokfelt, U., Garneau, M., Charman, D.J., Holden, J., 2015a. Evaluating the use of testate amoebae for palaeohydrological reconstruction in permafrost peatlands. Palaeogeogr Palaeocl Palaeoecol 424, 111-122.

Swindles, G.T., Holden, J., Raby, C., Turner, T.E., Blundell, A., Charman, D.J., Menberu, M.W., Kløve, B., 2015b. Testing peatland water-table depth transfer functions using high-resolution hydrological monitoring data. Quat Sci Rev 120, 107-117.

Tveito, O.E., Førland, E., Heino, R., Hanssen-Bauer, I., Alexandersson, H., Dahlström, B., Drebs, A., Kern-Hanssen, C., Jónsson, T., Vaarby Laursen, E., Westman, Y., 2000. Nordic Temperature Maps, p. 54.

Yang, Z.L., Hanna, E., Callaghan, T.V., Jonasson, C., 2012. How can meteorological observations and microclimate simulations improve understanding of 1913-2010 climate change around Abisko, Swedish Lapland? Meteorol Appl 19, 454-463.

Zuidhoff, F.S., Kolstrup, E., 2000. Changes in palsa distribution in relation to climate change in Laivadalen, northern Sweden, especially 1960-1997. Permafrost Periglac 11, 55-69.
